# Nanocomposite Hydrogel‐Based Optical Fiber Probe for Continuous Glucose Sensing

**DOI:** 10.1002/smsc.202300189

**Published:** 2023-12-03

**Authors:** Israr Ahmed, Said El Turk, Amal Al Ghaferi, Yarjan Abdul Samad, Haider Butt

**Affiliations:** ^1^ Department of Mechanical Engineering Khalifa University Abu Dhabi 127788 United Arab Emirates; ^2^ Department of Aerospace Engineering Khalifa University Abu Dhabi 127788 United Arab Emirates; ^3^ Cambridge Graphene Center University of Cambridge Cambridge CB3 0FA UK

**Keywords:** glucose sensing, hydrogels, nanocomposite, optical fiber

## Abstract

Diabetes mellitus (DM) presents a substantial global health concern due to elevated blood glucose levels, necessitating an affordable, rapid, and reliable continuous glucose monitoring (CGM) solution. In this pursuit, a pioneering approach is introduced utilizing optical fiber (OF) sensors based on nanocomposite photonic hydrogel functionalized with phenylboronic acid (PBA) for precise CGM. The fabrication of OF sensors involves a streamlined process, involving one‐step polymerization of PBA‐based hydrogel onto a commercial fiber tip and the integration of gold nanoparticles (AuNPs) via a simple dipping process. These sensors offer robust performance within the physiological glucose range (0–20 mm), exhibiting a remarkable 25% increase in transmission intensity and a 4 nm blue shift in the surface plasmon resonance with increasing glucose concentration. Additionally, there is a noticeable elevation in reflection intensity, affirming the sensor's suitability for remote sensing applications. These results are further validated using a green laser, underlining the method's reliability. The sensors exhibit a swift 30 s response time, followed by a 5 min saturation period, for all measurements. Practicality is demonstrated through smartphone readouts, utilizing the phone's photodiode to measure optical power changes concerning various glucose concentrations. These OF sensors hold great promise for CGM integration, enhancing diabetic management.

## Graphical abstract

This work presents PBA‐based hydrogel optical fiber sensors loaded with gold nanoparticles for continuous glucose monitoring applications. Sensitivity to various glucose concentrations is determined by analyzing changes in transmission and reflection intensities and surface plasmon resonance shift. The presented sensors demonstrate higher sensitivity, faster response time, and lower detection limits, complemented by smartphone readout methodology.

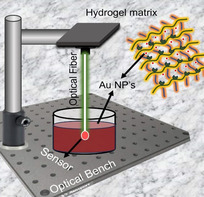

## 1 Introduction

Diabetes is a chronic medical condition caused by elevated blood glucose levels.^[^ ^1^, ^2^ ^]^ In the last few decades, there has been an astonishing growth in diabetic patients, making it a global health concern. According to the International Diabetic Federation, the total number of diabetic patients has reached 537 million in 2021.^[^ ^3^ ^]^ Also, diabetes is rising, especially in adults (20–79 years of age), making 10.5% of the adult population diabetic.^[^ ^4^ ^]^ Another point of worry is that almost half of these people do not know about their diabetic condition.^[^ ^3^ ^]^ Diabetes is not deadly, but it can lead to severe illness and complications, even death, if not handled properly.^[^ ^5^, ^6^ ^]^ The death toll from severe diseases caused by diabetes reached 6.7 million in 2021.^[^ ^4^ ^]^ It means diabetes is taking one human life every 5 s. Therefore, diabetic patients must monitor their blood glucose levels to avoid complications. So far, diabetes has no effective treatment and can be controlled only by proper diabetic management.^[^ ^7^ ^]^

One of the most common glucose measurement methods involves the finger prick technique, which requires patients to use this method 3–4 times a day to mitigate the risks associated with diabetes complications.^[^ ^8^ ^]^ This method carries the risk of infection and can be uncomfortable for patients. Therefore, companies like Abbott, Dexcom, Medtronic, and Senseonics have introduced advanced sensors for continuous glucose monitoring (CGM) applications. Many of these sensors are based on electrochemical principles and involve either invasive or minimally invasive approaches for glucose measurement.^[^ ^7^ ^]^ In contrast, noninvasive optical glucose sensors have attracted significant attention due to their potential to revolutionize glucose monitoring. Optical sensing, with its versatility encompassing methods like holographic, spectroscopic (near‐infrared, mid‐infrared, UV–vis, Raman, and photoacoustic), fluorescent sensing, and more, has emerged as a promising platform.^[^ ^9^, ^10^, ^11^ ^]^ Optical glucose sensors have garnered recognition for their user‐friendly, noninvasive design, enabling real‐time monitoring and the potential to reduce diabetes‐related complications.^[^ ^12^ ^]^

The optical fiber (OF) sensors have proven themselves as one of the most promising and reliable technologies. They have advantages such as low power consumption, small size, large bandwidth, higher sensitivity, better spatial resolution, and ease of operation.^[^ ^13^, ^14^ ^]^ These OF sensors can be combined with hydrogel materials for different sensing applications. Hydrogels are soft and biocompatible materials known for their molecular permeability.^[^ ^15^, ^16^ ^]^ Molecular permeability enables the functionalization of hydrogels with different stimuli‐sensitive materials. They can undergo a reversible change in their physical dimensions by swelling or contracting upon encountering external stimuli. Therefore, hydrogels are ideal candidates for different sensing applications, including pH,^[^ ^17^, ^18^ ^]^ temperature,^[^ ^19^, ^20^ ^]^ humidity,^[^ ^21^, ^22^ ^]^ alcohol,^[^ ^15^ ^]^ and glucose.^[^ ^7^, ^23^, ^24^, ^25^ ^]^

Hydrogel functionalized with phenylboronic acid (PBA) is one of the most common approaches for glucose sensing applications.^[^ ^26^, ^27^ ^]^ PBA is known for its affinity to bind with *cis*‐diol molecules present in glucose. This binding induces the reversible volumetric change in the hydrogel matrix.^[^ ^28^ ^]^ Therefore, PBA‐based hydrogel sensors can be exploited for different optical sensing approaches, including fluorescence,^[^ ^29^, ^30^, ^31^ ^]^ holographic,^[^ ^32^, ^33^ ^]^ and surface plasmon resonance (SPR) sensing.^[^ ^34^, ^35^ ^]^ These different approaches were explored to achieve higher sensitivity, faster response time, and lower detection limits. All these techniques have limitations in terms of sensitivity, responsiveness, reproducibility, limit of detection, sensor fabrication, and readout methodology. For example, Li et al. recently reported a fluorescence glucose sensor based on a functionalized hydrogel OF.^[^ ^31^ ^]^ The hydrogel was functionalized with fluorescein derivative CdTe QDs/3‐APBA. The hydrogel matrix swells because of the interaction between PBA and glucose molecules, which decreases the fluorescence of quantum dots. This decrease was recorded through OF to quantify glucose in the 0–20 mm concentration range. The sensors also showed an equilibrium time of 25 min.^[^ ^31^ ^]^

Similarly, Ahmed et al. reported a PBA‐based hydrogel sensor with nanostructure imprinted on the OF tip.^[^ ^36^ ^]^ Aztec nanostructures were introduced to enhance the sensitivity and detection limit of the sensors. The change in the zero and 1^st^ order diffraction of light was measured and correlated with the glucose concentration. The sensors showed a higher sensitivity of 1.2 μW mm ^−1^ in the 0–50 mm concentration range, a saturation time of 5 min, and a lower detection limit of around 3 mm.^[^ ^36^ ^]^ In another report, Guo et al. reported on a plasmonic hydrogel OF sensor. The sensor used OF made from a PBA‐based hydrogel matrix with plasmonic AuNPs.^[^ ^35^ ^]^ The swelling of the hydrogel matrix with increasing glucose concentrations modulates the SPR, which was recorded in transmission spectra and used to quantify glucose concentrations. The authors reported the effect of two different concentrations of AuNPs for glucose sensing. The sensors were tested in the concentration range of 0–40 mm, providing a higher sensitivity of −0.13 dB mm ^−1^ and a response time of 50 min.

In this study, PBA‐based hydrogel sensors were fabricated using a one‐step polymerization process. The glucose‐sensing gel was polymerized on the tip of commercial OF. AuNPs were synthesized using a simple chemical reduction, the Turkevich method. Later, these AuNPs were loaded into the polymerized fiber tip by a simple dipping method. The fabricated sensor was measured in different glucose concentrations (0–20 mm). Both transmission and reflection modes were employed to determine the sensing capability of the OF sensor. The change in the transmission and reflection spectra and the position of SPR was measured and correlated with the known glucose concentration. Higher sensitivity and rapid response time were recorded for these sensors. In the end, a smartphone was employed to record the change in the transmitted light with changing glucose concentrations to show the practicality of the fabricated sensor.

## 2 Experimental Section

### 2.1 Materials

Acrylamide (AA)—Monomer, N′N‐methylene bisacrylamide (MBAA)—crosslinker, 2,2‐Dimethoxy‐2‐phenyl‐acetophenone (DMPA)—photo‐initiator, 3‐(acrylamido) PBA—functionalizing material, dimethyl sulfoxide (DMSO) – solvent, D(+)‐ Glucose, Oxoid phosphate‐buffered saline tablets, sodium citrate dihydrate, and gold chloride (III) hydrate were bought from Sigma‐Aldrich and utilized without any additional purification.

### 2.2 Fabrication of Glucose‐Sensing Gel

The glucose‐sensitive gel of 5 mm monomer solution was prepared by mixing 78.5 mol% of AA, 1.5 mol% of MBAA, 2% w/v of DMPA, and 20 mol% of PBA (functionalization agent). DMPA was dissolved in 1 mL of DMSO solution using magnetic stirring for 15 min. Later AA, MBAA, and PBA were added to the DMAP solution and left on a magnetic stirrer until the powder dissolved fully. Finally, 1 mL of glucose‐sensitive gel was obtained.

### 2.3 Fabrication of AuNPs

AuNPs were synthesized using a commonly used citrate‐reduction Turkevich method with the 1:11 of gold precursor (gold chloride (III) hydrate) to reducing agent (sodium citrate dihydrate). The reducing agent was used in excess since sodium citrate is not only used to reduce the gold precursor chemically but also to stabilize the formed NPs. The solution was initially prepared by pouring 60 mL of deionized water (DI) water into a clean beaker. The reducing agent was weighed and dissolved in 1 mL of DI water and left on a magnetic stirrer (300 rpm) until fully dissolved. The gold precursor was weighed and dissolved in 1 mL of DI water and added to the prepared 60 mL beaker, forming a pale yellowish solution. The gold precursor solution was heated on a hotplate while being stirred at 700 rpm until it reached its boiling point at 100 °C. The reducing agent solution was then added to the boiling gold precursor solution. After a little while, the color starts changing from yellowish to pinkish, indicating the start of the nucleation process for the formation of AuNPs (Figure 2b ‐ inset).^[^ ^37^, ^38^, ^39^ ^]^ As time passed, the pinkish color was observed to be darkening, indicating the formation of relatively larger NPs. After 10 min, there will be no more change in the color since all the gold precursor should be reduced, and the NPs solution was left for cooling at room temperature and later stored in the refrigerator at 4 °C. The transmission and absorption spectra of these AuNPs can be seen in Figure 2b. At 518 nm, a distinct SPR can be observed.

### 2.4 Fabrication of OF Sensors

**Figure 1:**
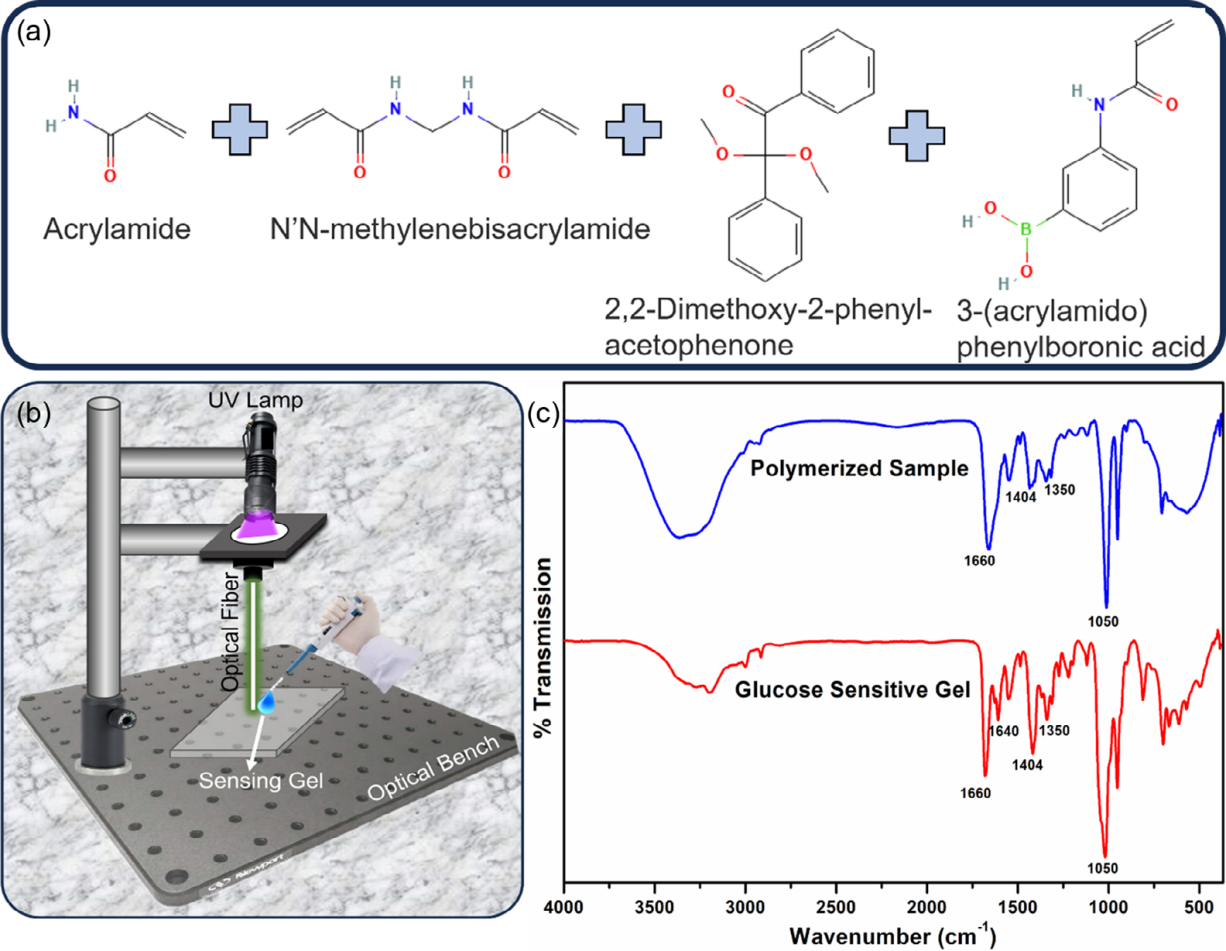
a) Chemical structure of materials used to fabricate glucose‐sensing gel. b) The fabrication process of OF sensor where UV light is transmitted through the same fiber to polymerize sensing gel on the fiber tip. c) FTIR spectra of glucose sensing gel before (red) and after polymerization (blue).

The schematic of the fabrication of OF sensor is illustrated in **Figure** **1**b. 15 μl of glucose sensing gel was drop cast on the tip of commercial OF. The UV light of 365 nm was coupled to the same OF, and the sensing gel was polymerized under UV light for 1 min. After 1 min, the UV light was turned off, and the phosphate buffer solution (PBS) solution was drop‐casted on top of the polymerized sensor so that the sensing gel could swell and detach from the glass slide.

### 2.5 Integration of AuNPs into OF Sensor

**Figure 2:**
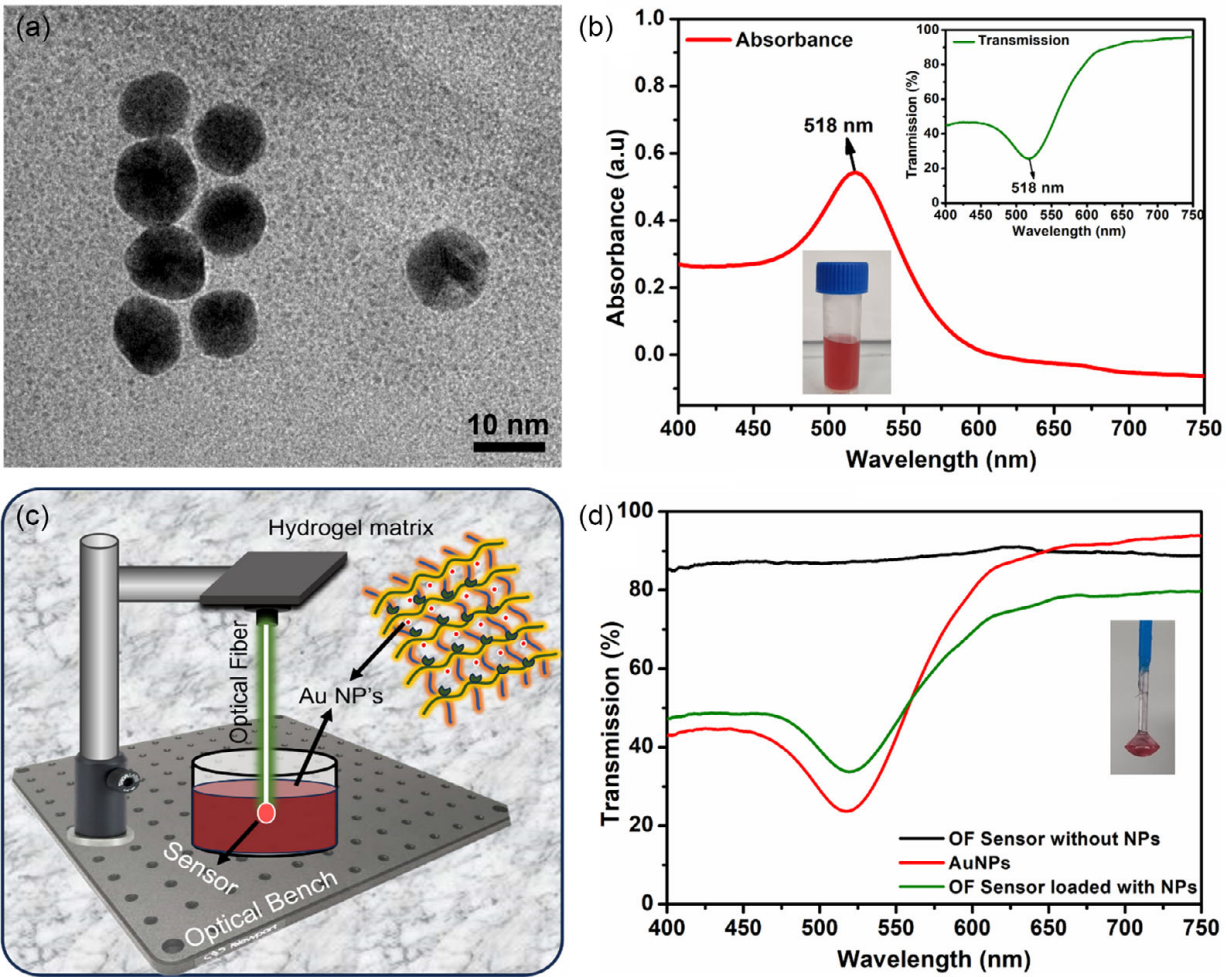
a) TEM image of AuNPs. b) The absorption spectrum of AuNPs suspended in DI water shows the SPR at 518 nm. The inset shows the transmission spectrum of the same NPs with SPR dip at 518 nm. c) Integration of AuNPs into PBA‐based hydrogel OF sensor. d) Comparison of transmission spectra of sensors without and with AuNPs along with the spectrum of solution of AuNPs.

OF sensor was immersed in the AuNPs solution to successfully integrate AuNPs into it (**Figure** **2**c). This allows the swelling of the hydrogel matrix, enabling the NPs to enter the sensors through the pores. After 1–2 h, the sensor's color changes from transparent to pink. The sensor was left inside the NP solution for 4 h, and then the transmission spectrum was measured, showing the SPR associated with AuNPs. Figure 2d inset shows the original image sensor loaded with AuNPs and attached to an OF.

### 2.6 Transmission Measurements

The transmission spectra of the OF sensor were measured using a transmission setup shown in Figure 4a. Transmitted light from the sensor was coupled into UV–vis‐spectrometer (Oceanview 2000+) via OF, and OceanArt software was used to translate this transmitted light into spectra. Transmission spectra were plotted in the visible range from 400 to 750 nm against the change in transmission percentage upon changing glucose concentration.

Transmitted optical power was also measured using the same measurement setup with some changes. The halogen lamp was replaced with a green laser (532 nm, Thorlabs), the spectrometer was replaced with a photodetector (S120C, Thorlabs), and the software was replaced with an optical power meter (PM100D, Thorlabs).

### 2.7 Reflection Measurements

Reflection spectra from the OF sensor were measured using the setup illustrated in Figure 4d. Three terminal OF bundle (RP21 – reflection probe, Thorlabs) was used to carry out these measurements. The reflected signal from the sensor at 90° was recorded, and reflection spectra were obtained. Reflected optical power using a green laser was also measured for different glucose concentrations.

### 2.8 Characterization Techniques

The morphological study of AuNPs is done using Tecnai transmission electron microscope (TEM). The morphological analysis of the glucose sensor loaded with AuNPs was performed using Nova Nano scanning electron microscope (SEM). The Fourier transform infrared (FTIR) spectra of the sensing gel with and without polymerization were carried out using PerkinElmer Spectrum 100. Swelling analysis was performed using a Zeiss optical microscope.

## 3 Results and Discussions

Glucose sensing gel was prepared by functionalizing hydrogel with PBA (Figure 1a). The detailed synthesis process can be found in the material and methods section. Later, this sensing gel was drop‐cast on the OF tip for polymerization (Figure 1b). FTIR measurements were performed using PerkinElmer Spectrum 100 to characterize the sensing gel and confirm the polymerization (Figure 1c). The FTIR spectra were measured in transmission mode with an average of 32 scans. The FTIR spectra of the sensing gel (red spectra) showed characteristic absorption bands C=O str. at 1660 cm^−1^ and C=C str. at 1640 cm^−1^. The peak at 1404 cm^−1^ represents the C‐N stretching in the acrylamide chain.^[^ ^40^, ^41^ ^]^ The functionalization of the hydrogel with PBA was confirmed by the presence of O‐B‐O at 1350 cm^−1^ and B‐O at 1015 cm^−1^, corresponding to PBA's characteristic absorption peak.^[^ ^42^, ^43^, ^44^ ^]^ The C=C (1640 cm^−1^) peak disappeared in the polymerized FTIR spectra, reflecting the successful polymerization process of the hydrogel matrix (blue spectra).

The synthesis of AuNPs was done using the well‐established Turkevich method. The TEM analysis of NPs showed spherical morphology and an average particle size of 10 nm. TEM image showing AuNPs can be seen in Figure 2a. The polymerized fiber tip was immersed in this AuNPs solution for 4 h to complete the integration process to finalize the sensor. The transparent sensor's color turns pinkish, showing the successful integration of AuNPs. The transmission spectrum of the OF sensor was measured to see the effect of SPR. Figure 2d presents the comparison of the transmission spectra of AuNPs suspended in DI water (red curve) and OF sensor (AuNPs loaded in hydrogel matrix – green curve). A slight redshift of 2 nm in the SPR can be observed for the OF sensor. This shift is attributed to the NPs moving closer to each other during the integration process. The interparticle spacing between NPs decreased when loaded inside the polymer matrix, resulting in a redshift of SPR.^[^ ^45^ ^]^ Also, the transmission spectrum of the OF sensor without NPs was measured and shown by a black curve. The transmission curve follows the straight‐line behavior with no dominant absorption feature, showing the transparency of the hydrogel matrix.

**Figure 3:**
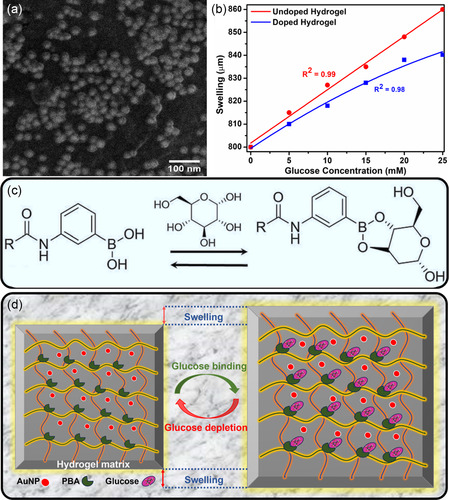
Comprehensive analysis of the characteristics and functions of the OF: a) SEM of the cross‐section of polymerized hydrogel matrix showing AuNPs distribution. b) Comparison of swelling of hydrogel matrix with and without AuNPs. c) Complexation between PBA and *cis*‐diol molecules present in glucose. d) Schematics of hydrogel matrix with AuNPs, PBA, and attachment of glucose to PBA changing the physical dimensions of hydrogel matrix by swelling.

High‐resolution SEM was employed to investigate the integration of AuNPs into a hydrogel matrix. The polymerized sample loaded with AuNPs was sectioned through the center to examine the sensor's cross‐section. The cross‐section surface of the sensor can be seen in **Figure** **3**a. A clear spherical morphology and similar particle size agree with the TEM results. These findings indicate the successful integration of AuNPs, providing evidence that they are indeed embedded within the hydrogel matrix. Additionally, distinct nanoparticles are clearly visible and show no signs of aggregation. The distinct dispersion of NPs within the sensor matrix contributes to the improved performance and effectiveness of the OF sensor. This dispersion allows for the optimal utilization of the unique properties of AuNPs, resulting in accurate and reliable sensing applications.^[^ ^46^ ^]^

The swelling analysis of samples with and without NPs was conducted under optical microscopy. A small disc of similar dimensions was prepared to investigate the swelling kinetics. Both samples showed an increased diameter with increasing glucose concentration (0–25 mm). The sample without NPs showed a linear increase in the diameter from 800 to 860 μm, showing an overall swelling of 60 μm, which agrees with the already published values.^[^ ^36^ ^]^ In contrast, the sample loaded with AuNPs showed a decrease in the swelling ratio (800 to 840 μm) compared to the sample without NPs (Figure 3b). This decrease can be associated with reduced bonding sites between PBA and *cis*‐diol molecules of glucose because of the presence of AuNPs. The interaction of PBA with glucose is vital for the hydrogel's responsiveness, and the presence of AuNPs may interfere with this binding by blocking certain binding sites, leading to a decrease in swelling at lower glucose concentrations.^[^ ^47^ ^]^ Furthermore, it was observed that, beyond a glucose concentration of 20 mm, the change in the swelling of the sample loaded with NPs becomes negligible. This effect is likely a result of the limited availability of binding sites due to the presence of AuNPs. These nanoparticles hinder further swelling of the hydrogel matrix, leading to glucose saturation, where the binding sites are essentially saturated and cannot accommodate additional glucose molecules. In contrast, the sample without NPs continues to exhibit a linear increase in swelling at concentrations of 25 mm and above, as it is not constrained by the presence of AuNPs and can freely interact with increasing glucose molecules.^[^ ^28^ ^]^

The PBA is known for its affinity to bind reversibly with the *cis*‐diol molecules in glucose solution. PBA is neutral at low pH values (pH < pKa) and exhibits trigonal configuration. There are two possibilities for PBA to interact with glucose molecules. In the first case, PBA forms a cyclic ester while interacting with *cis*‐diol molecules. In this case, the pKa of cyclic ester remains less than the pKa of PBA. Hydrogel ion is released, which helps form stable boronate anion. While in the second case (pH > pKa), a boronate ion is formed by donating a proton. The PBA configuration will change from trigonal to tetrahedral upon binding boronate ions with glucose molecules. The number of boronate ions increases with this 1:1 complexation, ultimately increasing the Donnan osmotic pressure. This increase in pressure causes the swelling of the hydrogel matrix. The complexation between the PBA‐based hydrogel and *cis*‐diol is depicted in Figure 3c. Also, the schematic of hydrogel with AuNPs, PBA binding sites, its interaction with glucose molecules, and changes in the physical dimensions caused by swelling can be seen in Figure 3d.

**Figure 4:**
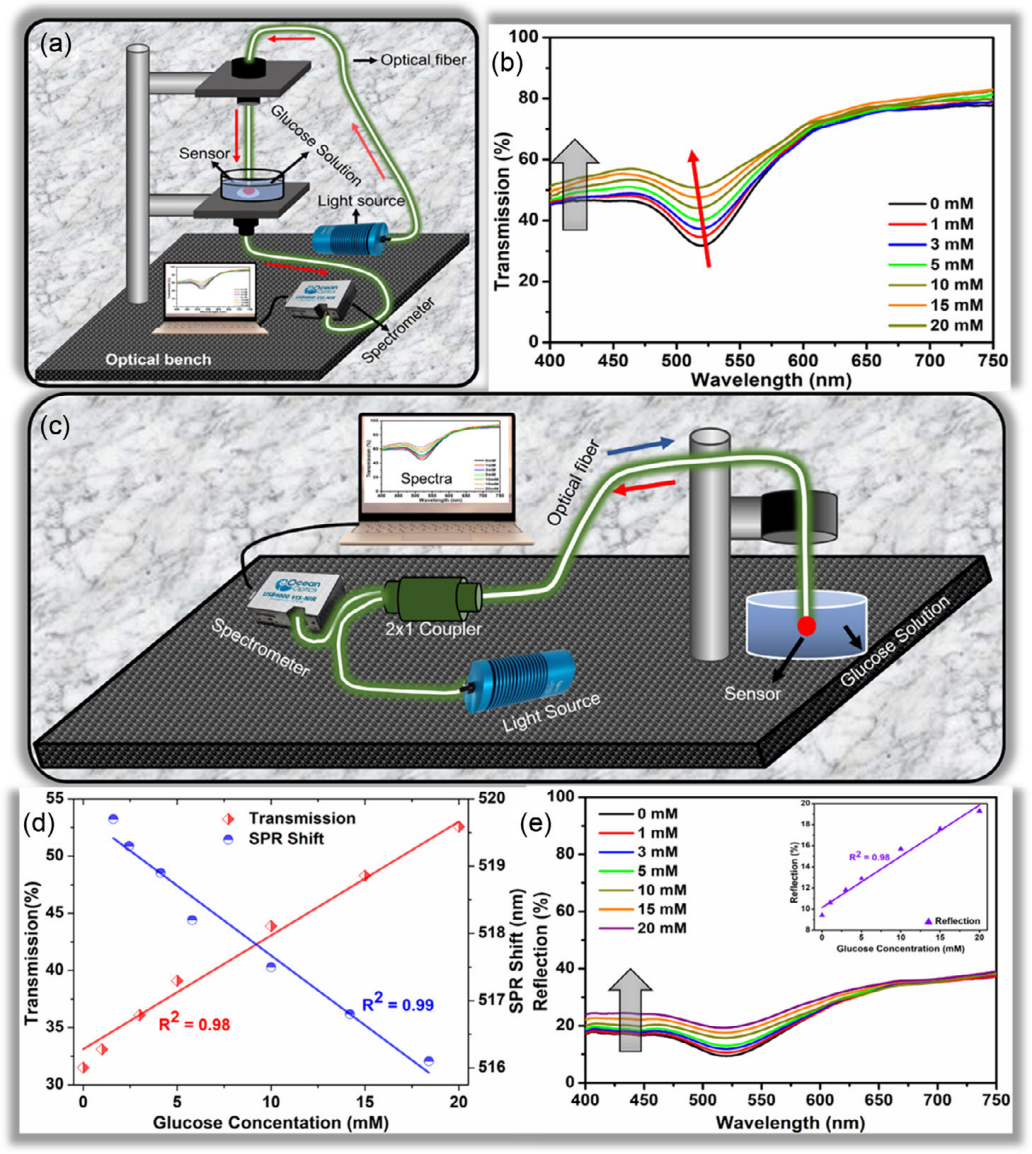
Experimental setup and analysis of the OF: a) Schematic of the transmission measurement setup. b) Transmission spectra of OF sensor at different glucose concentrations. c) Schematics of the reflection measurement setup. d) Quantitative analysis of transmission data showing the increase in transmission % and change in SPR position with changing glucose concentrations. e) Reflection spectra of OF sensor with increasing glucose concentrations. Inset shows the change in reflection % with changing glucose concentrations.

At first, the OF sensors were investigated using a transmission mode, considered the most practical mode for optical sensors. For this purpose, the OF sensor was connected to the broadband light source, and the transmitted light was collected through a spectrometer to get the transmission spectra in the visible wavelength range (400–750 nm). The schematic of the transmission setup can be seen in **Figure** **4**a. Different glucose concentrations ranging from 0 to 20 mm were tested. Prior to the transmission measurements, the OF sensor was immersed in PBS (0 mm) to achieve the saturation of hydrogel with water content as the result of hydrogen bonding. After that, the glucose concentration was changed to 1, 3, 5, 10, 15, and 20 mm and transmission spectra were recorded. A clear SPR associated with the AuNPs can be seen in Figure 4b. This SPR phenomenon is a characteristic feature of AuNPs and is an essential aspect of the sensor's performance. An increase in transmission is recorded with increasing glucose concentrations. The interaction between PBA and *cis*‐diol molecules in glucose solution results in an increasing number of boronate ions, which eventually increase the Donnan osmotic pressure inside the hydrogel matrix, changing the sensor's physical dimensions. The sensor's swelling and glucose concentration relationship are fundamental to its operation. It serves as the basis for the sensor's ability to provide accurate and timely measurements, making it a valuable tool for real‐time and continuous glucose monitoring. So, with increasing glucose concentrations, the sensor swells more. This swelling allows more light to pass through the sample.^[^ ^35^ ^]^ An overall increase of 22% in the transmission at the SPR position was recorded, giving a sensitivity of around 1.1% mm ^−1^ in the measured glucose concentration range of 0–20 mm (Figure 4d).

Besides increasing transmission, the swelling also affects the shape and position of the SPR. A blue shift of 4 nm in the SPR was recorded when glucose concentrations were changed from 0 to 20 mm, providing a sensitivity of 0.2 nm mm ^−1^. Also, a broadening of SPR can be observed with increasing glucose concentrations. The observed blue shift, increased transmission, and SPR broadening are associated with increased interparticle distance.^[^ ^17^ ^]^ The increase in the interparticle distance reduces the number of AuNPs positioned in the path of the transmitted light. This reduces the absorption due to AuNPs promoting the transmitted light, which is depicted as increased transmission (Figure 4b). This phenomenon is essential in the context of the sensor's functionality because it correlates with the sensor's ability to quantify variations in glucose concentrations. The interplay between interparticle distances, light transmission, and absorption forms the basis of the sensor's optical response, offering valuable insights into its performance for glucose sensing. The sensor demonstrates a superior response time of 30 sec and a saturation time of 5 min for all measurements. Also, complete reversibility over the multiple measurement cycles was also achieved.

For remote sensing applications, reflection mode was implemented to investigate OF sensors. For reflection measurement, a three‐terminal reflection fiber bundle was used. These three ends were connected to the light source, spectrometer, and OF sensor, as shown in Figure 4c. The reflection signal was recorded at an angle of 90° from the same fiber, and reflection spectra were recorded in the visible range (400–750 nm). The measured reflection spectra with different concentrations can be seen in Figure 4e. An increase in the reflected spectra is visible with increasing glucose concentration (0–20 mm) with the lowest measurable concentration of 1 mm. The reflection (%) changes from 9.4 to 19.3, providing a sensitivity of 0.5% mm ^−1^. The observed increase in reflection can be attributed to the swelling of the OF sensor. This swelling leads to a decrease in absorption from the AuNPs and, as a result, promotes both transmission and reflection of light. The broadening of the SPR dip in reflection mode poses a challenge when calculating changes in SPR. Again, the same response time of 30 s with a saturation time of 5 min was documented.

**Figure 5:**
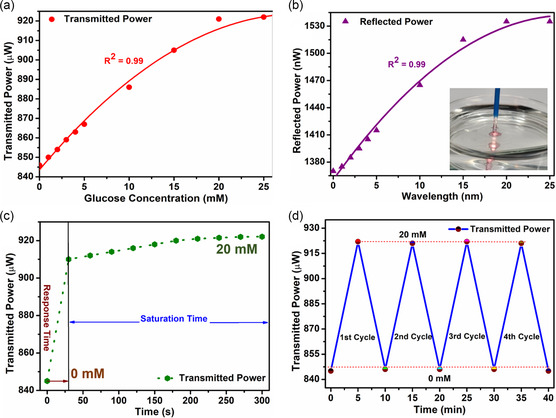
Experiment using green laser: a) Change in transmitted power with increasing glucose concentrations. b) Change in reflected power for different glucose concentrations. c) Time‐dependent transmission measurements for the calculation of response and saturation time of OF sensor for 0 to 20 mM glucose concentration. d) Demonstration of reusability of OF sensor for multiple cycles.

A green laser (λ = 532 nm) was employed to verify the transmission and reflection results as the SPR of the sensor corresponds to the green wavelength region, and maximum change in the transmission and reflection spectra was recorded in this region. The same measurement setup (Figure 4a) was used for transmission measurements, where the broadband light source was replaced with a green laser, a spectrometer with a photodetector, and software with an optical power meter. The power meter measures the optical power intensity with high accuracy. The green laser was coupled with the OF sensor, and output transmitted power was recorded. Before starting the experiment, the sensor was placed in the PBA solution (0 mm) for saturation. Later, the glucose concentrations were changed from 0 to 20 mm. An apparent linear increase in the transmitted power can be seen in **Figure** **5**a. The transmitted power changed from 846 to 922 μW when glucose concentration was changed from 0 to 20 mm, showing the sensitivity of the 3.75 μW mm ^−1^. The sensor showed a superior sensitivity compared to the other PBA‐based hydrogel OF sensors (**Table** **1**).^[^ ^28^, ^36^ ^]^ Higher glucose concentrations up to 30 mm were also tested, and no increase in the transmitted power was observed after 20 mm concentration, showing sensor saturation.

**Table 1:** Comparison of different glucose sensors with the current proposed sensor

Sensor type (PBA‐based)	Concentration range	Sensitivity	Response time	Limit of detection	Ref.
Diffraction Grating	1–200 mm	Change in diffraction angle was measured	10–15 min	10 mm	[32]
OF	1–12 mm	Swelling ratio: 0.5% mm ^−1^	20 min	1 mm	[48]
OF	1–50 mm	2.6 μW mm ^−1^	15 min	5 mm	[28]
OF	0–40 mm	–0.13 dB mm ^−1^	50 min	0.75 mm	[35]
Holographic sensor	0–9 mm	13.03 nm mm ^−1^	21−25 min	0.6 mm	[50]
Tapered OF	5–45 wt%	2032% RIU^−1^	–	5 wt%	[51]
OF SPR	0–22 mm	0.31 pm mm ^−1^	4 min	3 mm	[52]
PBA‐based hydrogel grating	0–50 mm	0.61% mm ^−1^	20 min	10 mm	[53]
Nanostructured OF sensor	0–50 mm	1.2 μW mm ^−1^	40 s	3 mm	[36]
Nanocomposite hydrogel‐based OF sensors	0–20 mm	3.75 μW mm ^−1^	30 s	1 mm	This work
		0.2 nm mm ^−1^			
		1.1% mm ^−1^			

The sensor was investigated for the calculation of response time along with the saturation of the transmitted power. The glucose concentration was changed from 0 to 20 mm, and the time from changing glucose level to the first response (increase in transmitted power) was calculated using a stopwatch. A sudden increase in the transmitted power was observed for the first 30 s. (Figure 5c) followed by a slow change in the transmitted power, reaching saturation in 5 min. For sensing applications, the reproducibility and reusability of the sensor play a vital role. The OF sensor was tested for multiple cycles to investigate the data's reusability, reliability, and reproducibility. The glucose concentration was changed from 0 to 20 mm for each cycle, and transmitted power was measured. At 20 mm, the hydrogel matrix swells because of the bonding between glucose and PBA, increasing optical transmitted power. For the second cycle, the glucose concentration was changed back to 0 mm, returning the transmitted power to its initial value. However, this value was slightly higher than the value of the first cycle because of some unwanted remains of glucose molecules attached to the sensor. The glucose sensor needs several washing cycles to remove these unwanted glucose molecules.^[^ ^36^ ^]^ Another solution to this problem is to reset the sensor in acetate buffer solution. The acetate buffer solution helps break the bonds between glucose and the PBA.^[^ ^35^ ^]^ This method requires immersing the sensor in a PBS buffer for 30 min before using it for a second time. Therefore, the OF sensor was washed with PBS solution to remove unwanted glucose and reused immediately. The sensor showed a complete reset to its initial value after washing cycles. Reusability over four measurement cycles is presented in Figure 5d.

The OF sensor was also tested in the reflection mode for remote sensing applications. Again, the same reflection setup (Figure 4c) was used with some changes. The light source was replaced with a green laser, the spectrometer was replaced with a photodetector, and the software was replaced with a power meter. The three‐terminal reflection fiber was connected to a laser, sensor, and photodetector. The increase in the reflected power was recorded with increasing glucose concentration. The AuNPs move apart because of hydrogel swelling, reducing absorption, and increasing transmission and reflection. The reflected power changed from 1370 to 1535 nW for glucose concentrations (0–20 mm). An overall change of 165 nW was recorded for the whole concentration range, providing a sensitivity of 8.25 nW mm ^−1^. The same response time of 30 sec and saturation time of 5 min was recorded for all measurements.

**Figure 6:**
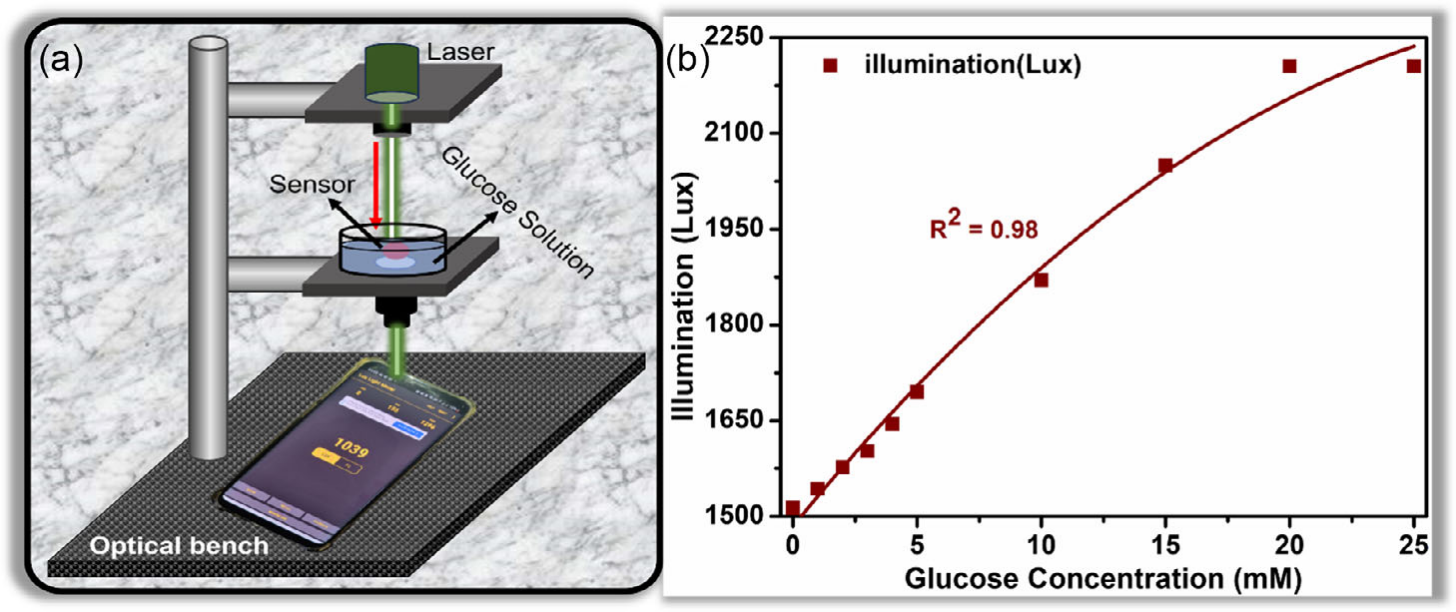
Illustration of smartphone methodology: a) Schematic of smartphone readout methodology. b) Illumination intensity (Lux) from OF sensor with increasing glucose concentration.

Besides all the advanced research in optical glucose sensors, one of the key challenges is the convoluted readout.^[^ ^24^, ^32^, ^34^, ^48^, ^49^ ^]^ Therefore, a smartphone was used to perform the readout to simplify the readout methodology for the proposed OF sensor. A smartphone's photodetector (ambient light sensor) was exploited to achieve this. A free application was downloaded from the Google Play Store and installed on the Android smartphone. The change in the illumination intensity was recorded in the unit of Lux. The OF sensor was coupled with the green laser, and transmitted light was recorded for different glucose concentrations (0–25 mm). The schematic of the measurement setup is depicted in **Figure** **6**a. The illumination is plotted against different glucose concentrations and can be seen in Figure 6b. A linear response was recorded for 0–20 mm glucose concentrations. The sensitivity of 34.6 Lux mm ^−1^ and the response time of 30 s. were recorded. No change was recorded when the glucose concentration was changed from 20 to 25 mm. The lowest limit of detection was measured as 1 mm. Also, no change in the Lux was measured for the concentration lower than 1 mm.

PBA‐based glucose sensors are well‐established and widely reported in the literature. The functionalization of hydrogel with PBA adds a new dimension to the optical glucose sensors because of the swelling parameter of the hydrogel. This swelling behavior of PBA‐based hydrogel was combined with the sensitivity of AuNPs. The SPR of AuNPs was used as the sensing unit. A slight change in the dimensions of the hydrogel matrix affects the SPR, which can be correlated with a change in glucose concentration. A comparison of the OF sensors proposed in this work with other glucose sensors is presented in Table 1. This table facilitates a straightforward evaluation of the proposed sensors’ superior attributes in terms of sensitivity, response time, and detection limit when contrasted with recently reported sensors.

### 3.1 Statistical Analysis

The spectroscopic data were obtained using a UV–vis spectrometer (USB2000+), and transmission and reflection spectra were recorded using OceanView software. The integration time was set to automatic, and an average of 20 spectra were obtained directly from the software. OrigionPro software was used to plot the data obtained from OceanView with any further processing. The OF sensor size prepared for all measurements was ≈500 μm.

## 4 Conclusion

A nanocomposite hydrogel‐based OF sensor was fabricated using a simple facial process. AuNPs were loaded into a PBA‐based hydrogel matrix. The change in SPR that emerged from hydrogel swelling was recorded and correlated with glucose concentrations. The sensor was tested in transmission and reflection modes for glucose concentrations within the physiological range of healthy (3.9–6.9 mm) and diabetic patients (7.2–10 mm). A green laser was also employed to verify transmission and reflection results. The proposed sensors showed superior sensitivity, rapid response time (30 s), and a detection limit of 1 mm. Complete reusability and reproducibility of data over multiple measurement cycles were recorded, which is essential for real‐time and continuous glucose monitoring applications. Most importantly, the smartphone readouts were enabled to show the practicality of sensing.

## Conflict of Interest

The authors declare no conflict of interest.

## Data Availability

Data sharing is not applicable to this article as no new data were created or analyzed in this study.
